# Factors associated with anemia among reproductive age women in Nigeria; evidenced by the Nigeria malaria indicators survey: spatial and multilevel model analysis

**DOI:** 10.1186/s40834-024-00275-x

**Published:** 2024-04-02

**Authors:** Gosa Mankelkl, Beletu Kinfe

**Affiliations:** 1 Department of Biomedical Sciences, College of Medicine and Health Science, Wollo University, Dessie, Ethiopia; 2 Department of occupational Health and safety, College of Medicine and Health Science, Wollo University, Dessie, Ethiopia

**Keywords:** Anemia, Multilevel, Spatial, Nigeria

## Abstract

**Background:**

Anemia is a global public health problem among women of reproductive age group, especially in developing countries, which affect health, social and economic development that result in low physical activity, increased maternal morbidity and mortality and adverse neonatal outcome especially those with severe anemia. However, there is limited reliable and updated data on the spatial variations of anemia and its associated factors among reproductive-age women in Nigeria.

**Methods:**

Secondary data analysis was conducted using data from the recent Nigeria malaria indicators survey datasets. The study comprised a total of 14,476 reproductive-age women. Spatial and multilevel mixed effect analysis on determinants factors of anemia among reproductive age women in Nigeria evidenced by the recent Nigerian malaria indicators survey. Finally, the percentage and odd ratio, its 95% confidence intervals, and the result of spatial analysis were reported.

**Result:**

This study includes a total weighted sample of 14,476 reproductive-age women from the Nigeria malaria indicators survey. The prevalence of anemia was 24.6% in Ethiopia. Being between the age range of 30–34 years [AOR: 0.217, 95% CI (0.171, 0.274)], Attending higher education [AOR: 0.848, 95%CI (0.740, 0.972)] and being male headed household [AOR: 0.540, 95% CI (0.471, 0.620)] were protective for anemia. On the other hand being poorest [AOR: 1.542 95%CI (1.299, 1.830)] and being listening radio less than once a week [AOR: 1.013, 95% CI (0.908, 1.131)] were risk for anemia.

**Conclusion:**

In this study Individual level factors were associated with anemia and also there were spatial variations in anemia across the region among reproductive-age women. Empowering women to have better educational status, improving the wealth index, and promoting education about prevention and control strategies of anemia through media especially in developing regions were the key factors to reduce anemia among reproductive age women in Nigeria.

## Background

Anemia is characterized by a drop in red blood cell count or hemoglobin level per unit volume in the blood below normal range, making the oxygen-carrying ability of red blood cells (RBCs) insufficient to meet the body’s physiologic needs [1]. Based on hemoglobin levels, anemia in women of reproductive ages (WRAs) who are not pregnant can be classified as mild (11–11.9 g/dl), moderate (8–10.9 g/dl), or severe (8 g/dl) [2]. Similar to this, the severity of anemia can be categorized as a severe, moderate, or mild public health issue when the prevalence is greater than 40%, 20–39%, and 5–19%, respectively, and is considered to be of public health significance if the prevalence is 5.0% and higher [3].

Since it is a cheap, simple, and accurate way to measure RBC concentrations during testing, hemoglobin (Hgb) plays a vital role in the diagnosis of anemia [4]. When hemoglobin concentration falls, the blood’s ability to carry oxygen is compromised, leading to persistent fatigue and other health problems [5]. The most typical symptoms of anemia are edema, headaches, low blood pressure, pallor, and low blood pressure. Furthermore, a particular kind of anemia may have general clinical characteristics that are specific to it [6].

Globally, about one-third (30%) of WRA are anemic, with major consequences for health, social and economic development and associated with an increased risk of morbidity and mortality [7], that is occurring at all stages of the life cycle [8]. According to estimates, the prevalence of anemia is 43% in developing nations and 9% in developed countries, respectively [9]. In the majority of sub-Saharan African nations, anemia prevalence is greater than 50% [10]., and adversely affect cognitive and motor development and cause fatigue and low productivity [11]. Similarly, it affects nervous system, respiratory and circulatory system, skin mucous membrane, digestive system, endocrine system [12]. Anemia is a sign of poor nutrition and health, and it raises the risk of complications during pregnancy, including miscarriage, stillbirth, preterm, and low birth weight [13].

Anemia has multi-factorial causes, iron deficiency largely due to inadequate dietary intake of iron, poor absorption, and period of life where iron requirements become high [11]. Due to their increased iron requirements during growth, pregnancy, lactation, menstrual blood loss, and dietary deficits throughout their reproductive cycle, it is frequent among women of reproductive age [14]. Iron deficiency is thought to be the cause of roughly 50% of anemia worldwide, but the percentage likely varies among demographic groups and regions depending on the local circumstances [9]. Micronutrient deficiencies like vitamin A, vitamin B12, folic acid, riboflavin, and copper increase the risk of anemia. Other non-iron deficiency causes of anemia include acute and chronic infections, parasitic infections, malaria, and inherited or acquired disorders that affect hemoglobin synthesis and red blood cell production/survival [15–17].

Therefore, it is challenging to design preventative and control strategies of anemia to reduce the incidence of anemia among women of reproductive age due to the absence of updated and reliable data on spatial variations and determinant factors of anemia. Therefore, the purpose of this study is to identify the geographic variations in anemia and the contributing factors among Nigerian women of reproductive age. The result of this study was intended to help stakeholders (the Ministry of Health) in designing interventions to reduce anemia among women of reproductive age in collaboration with other stakeholders.

## Methods and materials

### Study setting and period

The 2021 NMIS is the third malaria indicator survey conducted in Nigeria. The 2021 Nigeria Malaria Indicator Survey (NMIS) was implemented by the NMEP in collaboration with the National Population Commission (NPC) and the National Bureau of Statistics (NBS), with technical assistance from ICF [18].

### Data source/extraction

After permission was secured through an online request by explaining the aim of the study, the data were taken from the Measure Demographic and Health Surveys (DHS) website (https://dhsprogram.com/data/dataset_admin/index.cfm).

### Study design

The community based cross-sectional study design was employed. A two-stage sampling strategy was adopted for the 2021 NMIS. In the first stage, 568 EAs were selected with probability proportional to the EA size. The result was a total of 568 clusters throughout the country, 195 in urban areas and 373 in rural areas. Complete listing of households in these clusters was conducted between 26 August and 18 September 2021, with the resulting lists of households serving as the sampling frame for the selection of households in the second stage. GPS dongles were used to capture coordinates during household listing in the 2021 NMIS sample clusters. In the second stage’s selection process, 25 households were selected in each cluster via equal probability systematic sampling [18].

### Study population

The Woman’s Questionnaire was used to conduct interviews with all women aged 15 to 49 who were either visitors or long-term residents of the selected households who were there the night before the study [18]. 14,476 women had interviews conducted with them. 9835 of the 14,476 women who were interviewed were from a rural area, while 4641 were from an urban area. Since the outcome variable for this study was anemia status among reproductive age women so, the final sample size for this analysis was 14,476.

### Study variables

**The outcome variable** for this study was the anemia status, which was coded as “0” if the women were anemic (mild, moderate and severe anemia) and “1” if the women were not anemic.

**Individual-level variable**: maternal age, educational level, sex of household head, wealth index, sources of drinking water, types of toilets facility, frequency of listening to radio, and frequency of watching television.

**Community-level variable**: place of residence.

### Data management and analysis

In all the analysis, we adjusted for the complex nature of the survey design by accounting for clustering, stratification, and weighting. Due to the comparisons and combination (pooled data) of surveys from different regions, with different target population sizes, the weights were normalized. This was done by dividing the women’s standard weights and their total number the country by the respective survey sampling fraction. Data Extraction, recoding, and both descriptive and analytical analysis were carried out using STATA version 14 software. The multilevel analysis was fitted due to the hierarchical nature of the Malaria Indicator Survey data. In this study, the multilevel mixed-effects model was employed and the dependent variable was binary.

The Interclass Correlation Coefficient (ICC) was used to evaluate the regional variability. In order to choose variables for multivariate analysis, bivariate analysis was first performed on the following variables: maternal age, place of residence, educational status, sex of household head, wealth status, sources of drinking water, types of toilet facilities, frequency of listening to radio, and frequency of watching television. Only variables with *p*-values less than 0.05 were taken into consideration for multivariate analysis.

### Spatial analysis

The weighted frequency of anemia, the cluster number, and the geographic coordinates were integrated in Stata 14. After that, data was exported to Excel and then imported for spatial analysis into ArcGIS 10.3.

### Spatial autocorrelation analysis

The spatial autocorrelation (Global Moran’s I) statistic examines the distribution of anemia among women of reproductive age in Nigeria. Moran’s I is a spatial statistic that uses the entire data set to generate a single output value that varies from − 1 to + 1 in order to evaluate spatial autocorrelation. I, Moran’s Values around − 1 suggest scattered anemia, whereas values near + 1 indicate clustered anemia, and values near 0 indicate random distribution of anemia. A statistically significant Moran’s I (*p* < 0.05) lead to the failure to reject the alternative hypothesis and rejection of the null hypothesis (anemia is randomly distributed) and indicates the presence of spatial autocorrelation.

### Hot spot analysis (Getis-OrdGi* statistic)

Getis-OrdGi* statistics were used to generate the GI* statistics for each region to determine how the spatial autocorrelation varies in Nigeria. To determine the statistical significance of clustering, the *p*-value is assessed for significance using the Z-score. A “hot area” is suggested by high GI* statistical output, whereas a “cold spot” is suggested by low GI* statistical output.

### Spatial interpolation

To determine the impact of a particular event throughout the country, it is highly expensive and time-consuming to gather trustworthy data. As a result, using the observed data, interpolation was utilized to estimate a portion of a certain area. The spatial interpolation approach was used to forecasts anemia in the unstudied portions of the country based on sampled EAs from MIS. In this work, anemia in unobserved regions of Nigeria was predicted using the standard Kriging spatial interpolation approach. The burden of anemia in non-sampled regions was estimated for this study using the standard Kriging approach.

### Ethical consideration

Since the Malaria Indicator Survey program used secondary, easily accessible survey data, ethical review and participant consent were not necessary for this particular study. DHS Program granted us permission to utilize data from their website that we had obtained after requesting that we do so.

### Socio-demographic characteristics of the participants

A total of 14,476 reproductive age women were involved in this study. About 2793(19.3%) were found between the age ranges of 15–19 years old. The majority of respondents 9835 (67.9%) were live in rural area, 8470(58.5%) were Muslim, 5156(35.6%) were not attending formal educations, 12,573(86.9%) were male headed households, 2651(18.3%) were poorest, 4804(33.2%) had unimproved water sources, 5586(38.6%) has unimproved toilets facility, 8168(56.4%) not listening radio at all, 7802(53.9%) not watching television at all and 3559(24.6%) were anemic (severe anemia (1.6%), moderate anemia (12.5%) and mild anemia (10.4%)) (Table 1).

**Table 1:** Socio demographic characteristics of reproductive age women in Nigeria 2024

Characteristics	Categories	Anemia		Frequency	Percentage
		Yes	No		
Age group	15–19	142	2651	2793	19.3
	20–24	559	1904	2464	17.0
	25–29	964	1696	2660	18.4
	30–34	836	1526	2362	16.3
	35–39	655	1309	1964	13.6
	40–44	312	1108	1420	9.8
	45–49	91	723	814	5.6
Place of residence	Urban	1154	3488	4641	32.1
	Rural	2406	7429	9835	67.9
Religion	Catholic	272	786	1057	7.3
	Other Christian	1207	3685	4892	33.8
	Islam	2063	6407	8470	58.5
	Traditionalist	18	37	54	0.4
	Other	0	3	3	0.0
Educational status	No education	1396	3760	5156	35.6
	Primary	504	1585	2089	14.4
	Secondary	1170	4193	5364	37.1
	Higher	489	1378	1867	12.9
Sex of house hold head	Male	3258	9315	12,573	86.9
	Female	302	1602	1903	13.1
Wealth index	Poorest	606	2046	2651	18.3
	Poorer	687	2043	2730	18.9
	Middle	661	2138	2799	19.3
	Richer	727	2279	3006	20.8
	Richest	878	2411	3289	22.7
Sources of water	Improved	2388	7284	9672	66.8
	Unimproved	1171	3633	4804	33.2
Types of toilets	Improved	2187	6703	8890	61.4
	Unimproved	1372	4214	5586	38.6
Frequency of listening radio	Not at all	1955	6213	8168	56.4
	Less than once a week	766	2327	3093	21.4
	At least once a week	838	2378	3216	22.2
Frequency of watching television	Not at all	1885	5917	7802	53.9
	Less than once a week	615	1837	2451	16.9
	At least once a week	1059	3163	4223	29.2
Level of anemia	Severe	230	0	230	1.6
	Moderate	1817	0	1817	12.5
	Mild	1513	0	1513	10.4
	Non-anemic	0	10,917	10,917	75.4
Anemia status	Anemic	3559	0	3559	24.6
	Not anemic	0	10,917	10,917	75.4

### Spatial analysis results

#### Spatial distribution of anemia

**Fig. 1:**
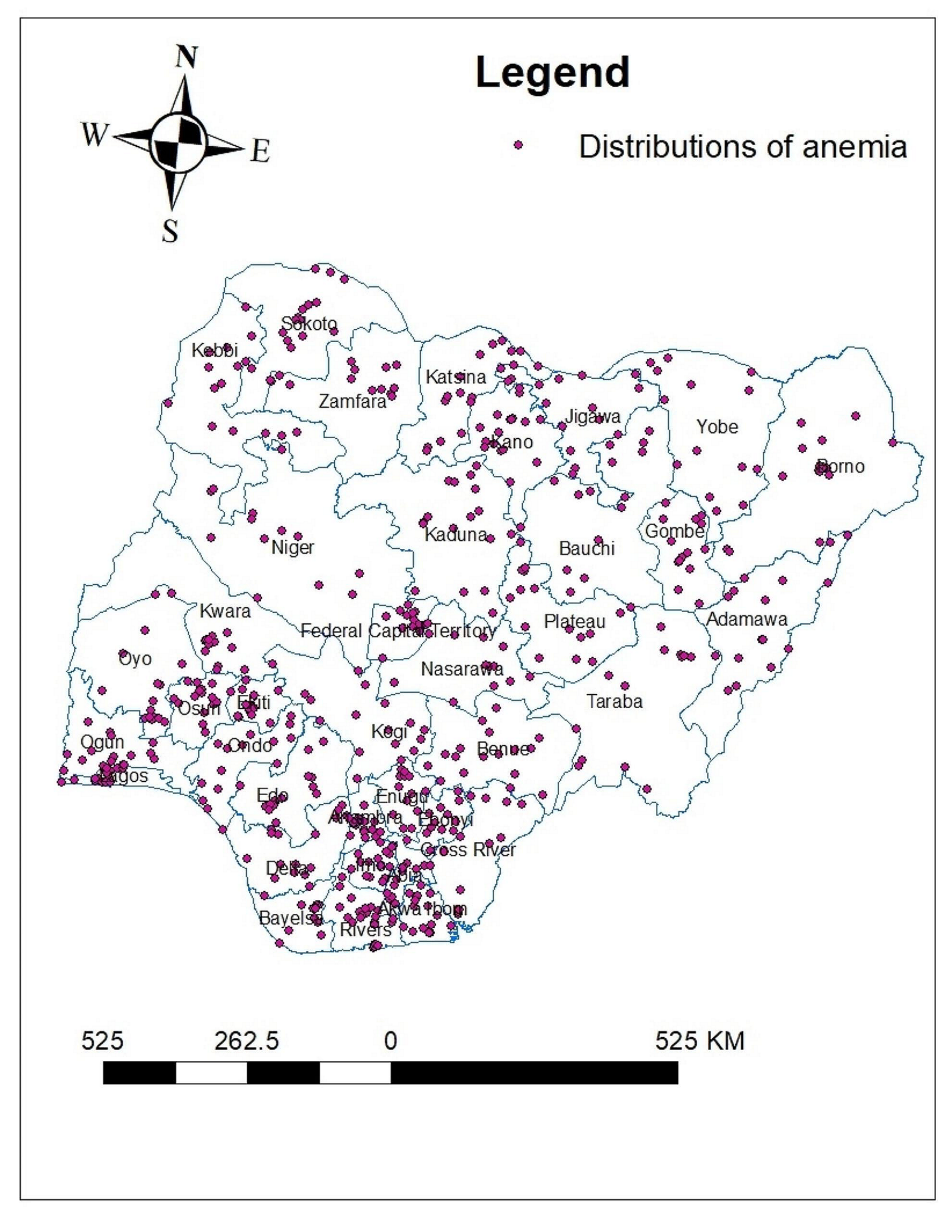
Distributions of anemia among reproductive age women in Nigeria 2024

In Nigeria, anemia status was analyzed geographically using 568 clusters. The number of anemia instances in each cluster corresponds to one enumeration area at each spot on the map. This study’s analysis of the spatial distribution of anemia showed that a higher proportion of anemia in southern and south west region of Nigeria. The northern, Western and north east region of Nigeria had a low of proportion of anemia (Fig. 1).

#### Spatial autocorrelation anemia

**Fig. 2:**
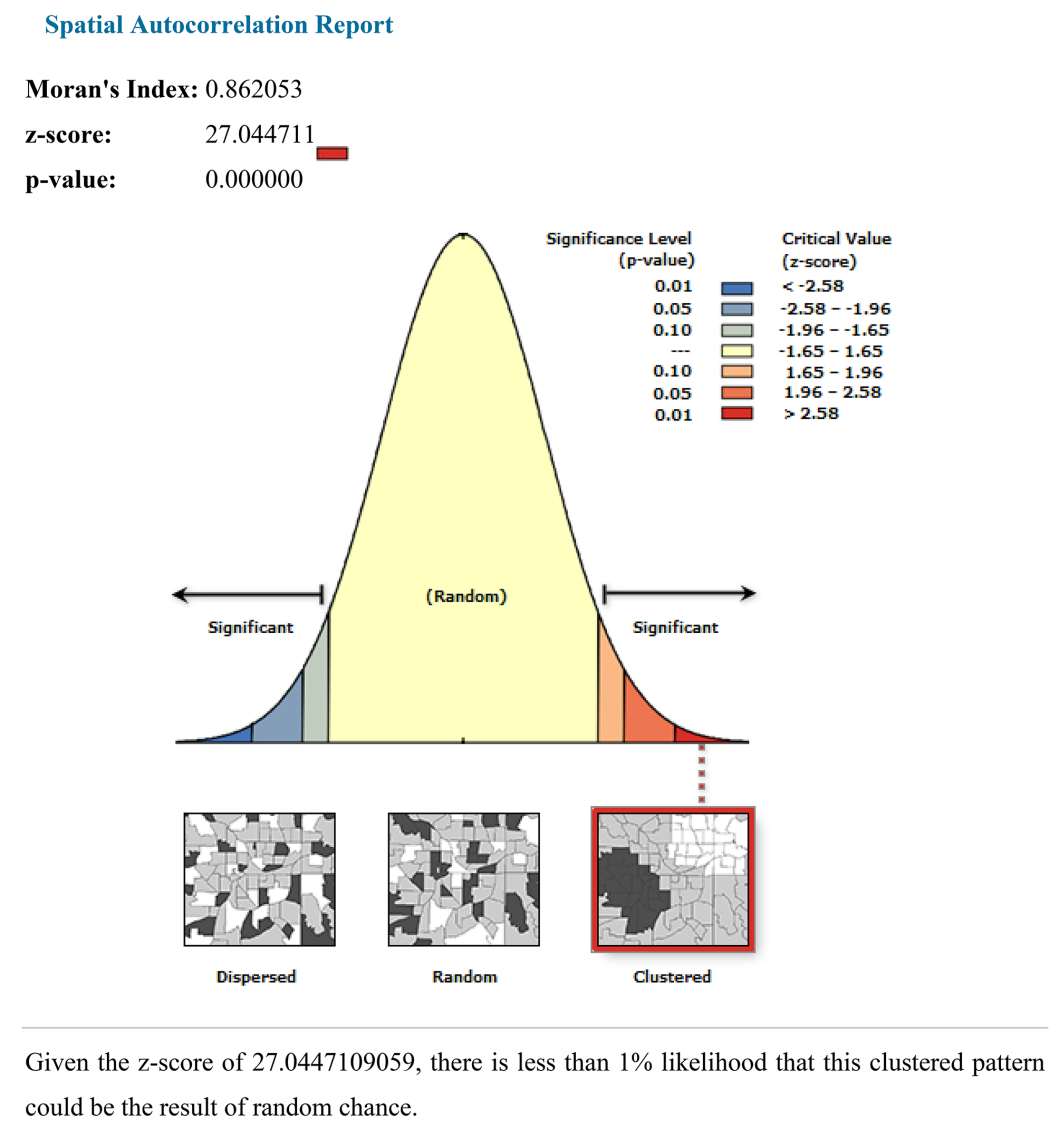
Autocorrelations of anemia among reproductive age women in Nigeria 2024

The spatial autocorrelation result reveals whether anemia in Nigeria is randomly distributed across the region, clustered, or dispersed. The results of the spatial autocorrelation study showed a clustering effect in the anemia across the country. The clustered patterns (on the right’s red box side) demonstrated a clustering effect on the anemia in Nigeria. The outputs have automatically generated keys on the right and left sides of each panel. The probability that this clustered pattern is the result of random chance is less than 1%, according to the z-score of 27.04 (*p*-value < 0.001). The bright red and blue colors to the end tails indicate an increased level of significances.(Fig. 2).

#### The hotspot analysis result

**Fig. 3:**
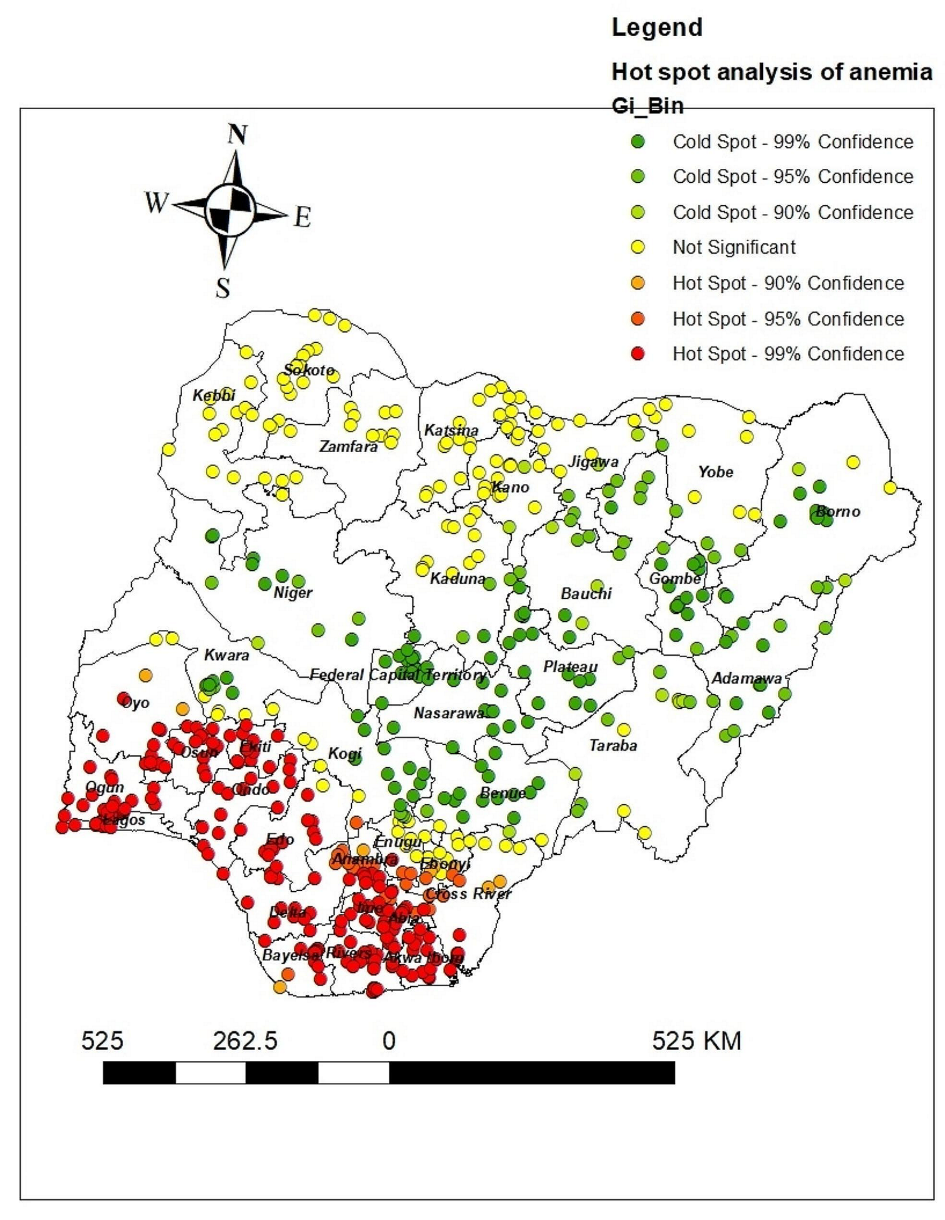
Hot spot analysis of anemia among reproductive age women in Nigeria 2022

The hotspot analysis result shows the high proportion (hotspot) and low proportion (cold spot) areas of anemia in Nigeria. The red colors were seen in the Lagos, Ogun, Osun, Oyo, Ondo, Edo, Ekiti, Bayelsa, Delta, Rivers, Akwa Ibom, Abia, Imo, Rivers, Anambra, Enugu, Ebonyi, and Cross River which are hot spot areas (areas with high proportion of anemia). The green-colored were cold spots (areas with a low percentage of women with anemia) were found in Borno, Adamawa, Gombe, Bauchi, Plateau, Nasarawa, Benue, Federal Capital Territory, and Kogi (Fig. 3).

#### Spatial interpolation or prediction

**Fig. 4:**
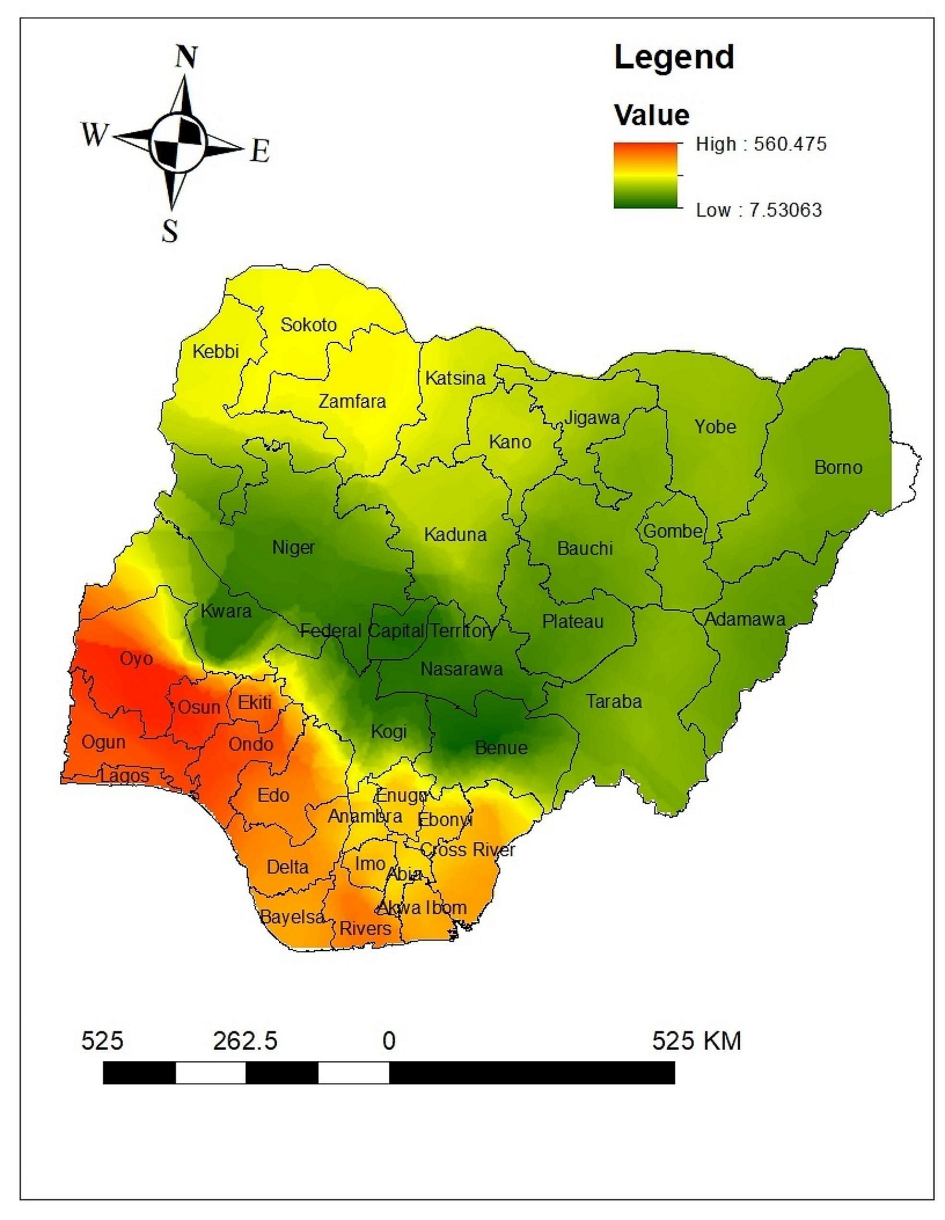
Interpolations of anemia among reproductive age women in Nigeria 2024

Based on the sampled region, the spatial interpolation approach predicts the proportion of anemia for non-sampled areas. The area map was described using the standard Kriging method. The green color represents the projected low prevalence of anemia. If the area’s color shifted from green to red, it indicates that more people in the area are anemic than was previously expected. The country is predicted to anemia at a low prevalence, as shown by the green color. According to the green color prediction’s results Jigawa, Yobe, Borno, Adamawa, Gombe, Bauchi, Katsina, Kaduna, Kano, Plateau, Taraba, Nasarawa, Benue, Federal Capital Teriteri, Niger, Kogi, Kwara and Kogi had low prevalence of anemia. The red color prediction showed that the regions of Lagos, Ogun, Osun, Oyo, Ondo, Edo, Ekiti, Bayelsa, Delta, and Rivers had the higher prevalence of anemia nationwide (Fig. 4).

### Model comparison

Four models were built for this multistage investigation. The first model was built. Without independent factors, it is possible to determine how community variation affects women’s anemia status. The second model included variables at the individual level. Community level characteristics were incorporated in the third model. Finally, the fourth model took into account factors at both the individual and community levels. The ICC in the null model showed that among women of reproductive age, there was a variance in anemia status of 7.12% in the communities. The variance in anemia status among women of reproductive age is described by variables at the individual level in 8.28% of occurrences. The difference in anemia status among women of reproductive age is accounted by community level variables were 11.52%. In the end, 14.69% of the variances among women in reproductive age were caused by variables at the individual and community levels. Deviance was used to evaluate model fitness for model comparison (AIC). As a result, it was determined that Model IV, which included factors at both the individual and community levels and had the lowest deviance (AIC) value, provided the best fitted model. Variables having a *p* < 0.05 significance levels were considered to be significant predictors of anemia status among reproductive-age women (Table 2).

**Table 2:** Revealed the random effect of anemia and model comparison

Parameters	model I	model II	model III	Model IV
ICC (%)	7.12	8.28	11.52	14.69
Model fitness				
**AIC**	4256	4189	3006	2968

### Factors analysis associated with anemia

In bivariate logistic regression analysis, all variables were evaluated to find an appropriate independent variable for multivariate logistic regression analysis. For the final model, variables with bivariate logistic regression analysis *P* values less than 0.05 were taken into consideration. Age groups, places of residence, educational level, sex of household head, wealth index, source of drinking water, types of toilets facility, frequency of listening to radio, and frequency of watching television were all taken into account in the bivariate analysis. The results of the bivariate analyses showed that among women of reproductive age, anemia was statistically significantly associated with age groups, educational status, sex of household head, wealth index, and frequency of radio listening.

The multivariable logistic regression analysis also revealed that age groups, educational status, sex of household head, wealth index and frequency of listening radio were significantly associated with anemia among reproductive age women.

The finding from this study shows that the odd of anemia between the age range of 30–34 years old were 0.217 less likely [AOR: 0.217, 95% CI(0.171,0.274)] compared to women whose age were between 15 and 19 years olds. The odd of anemia among reproductive age women who were attending higher education were 0.848 less likely [AOR: 0.848, 95%CI (0.740, 0.972)] relative to women who were not attending educations. The odd of anemia among male headed households were 0.540 times less likely [AOR: 0.540, 95% CI (0.471, 0.620)] compered to female headed households. The odd of anemia of anemia among poorest women were 1.542 more likely [AOR: 1.542 95%CI (1.299, 1.830)] relatives to richest. The odd of anemia among women who were listening radio less than once a week were 1.013 times more likely [AOR: 1.013, 95% CI (0.908, 1.131)] compared to women who were listening radio at least once a week (Table 3).

**Table 3:** Bivariate and multivariate analysis of factors associated with anemia among reproductive age women in Nigeria 2024

Characteristics	Categories	Anemia		COR with 95% CI	AOR with 95% CI
		Yes	No		
Age group	15–19	142	2651	1	1
	20–24	559	1904	2.338(1.774,3.081)***	2.248(1.698,2.975) ***
	25–29	964	1696	0.427(0.337,0.542) ***	0.416(0.327,0.529) ***
	30–34	836	1526	0.221(0.175,0.278) ***	0.217(0.171,0.274) ***
	35–39	655	1309	0.229(0.181,0.290) ***	0.228(0.180,0.288) ***
	40–44	312	1108	0.251(0.198,0.318) ***	0.249(0.197,0.317) ***
	45–49	91	723	0.445(0.346,0.573) ***	0.442(0.343,0.569) ***
Place of residence	Urban	1154	3488	0.979(0.903,1.062)	
	Rural	2406	7429	1	
Educational status	No education	1396	3760	1	1
	Primary	504	1585	0.956(0.848,1.078)	0.672(0.564,0.799) ***
	Secondary	1170	4193	1.115(0.966,1.288) **	0.807(0.679,0.960) **
	Higher	489	1378	1.271(1.125,1.435) ***	0.848(0.740,0.972) **
Sex of house hold head	Male	3258	9315	0.538(0.473,0.613) ***	0.540(0.471,0.620) ***
	Female	302	1602	1	1
Wealth index	Poorest	606	2046	1.230(1.092,1.385) ***	1.542(1.299,1.830) ***
	Poorer	687	2043	1.082(0.964,1.215)	1.293(1.102,1.516) **
	Middle	661	2138	1.178(1.049,1.324) **	1.279(1.107,1.478) ***
	Richer	727	2279	1.142(1.019,1.279) *	1.134(0.998,1.289) *
	Richest	878	2411	1	1
Sources of water	Improved	2388	7284	0.983(0.907,1.066)	
	Unimproved	1171	3633	1	
Types of toilets	Improved	2187	6703	0.998(0.923,1.079)	
	Unimproved	1372	4214	1	
Frequency of listening radio	Not at all	1955	6213	1.120(1.020,1.230)	1.038(0.922,1.169)
	Less than once a week	766	2327	1.070(0.955,1.199)**	1.013(0.908,1.131) *
	At least once a week	838	2378	1	1
Frequency of watching television	Not at all	1885	5917	1.051(0.964,1.147)	
	Less than once a week	615	1837	1.001(0.892,1.122)	
	At least once a week	1059	3163	1	

COR = crudes odds ratio, AOR = adjusted odds ratio; CI-confidence interval

Statistically significant at *P < 0.05; **P < 0.01; ***P < 0.001

## Discussions

The study assessed the spatial distribution of anemia and the factors that influence it’s among women of reproductive age in Nigeria, using the recent Nigeria malaria indicator survey. In general, this study showed geographical variations in the distributions of anemia among women of reproductive age in Nigeria. This study demonstrated that in the Lagos, Ogun, Osun, Oyo, Ondo, Edo, Ekiti, Bayelsa, Delta, Rivers, Akwa Ibom, Abia, Imo, Rivers, Anambra, Enugu, Ebonyi, and Cross River which were hot spot areas (areas with high proportion of anemia). On the other hand, this study showed that cold spots areas (areas with a low percentage of women with anemia) were found in Borno, Adamawa, Gombe, Bauchi, Plateau, Nasarawa, Benue, Federal Capital Territory, and Kogi. This finding was supported by the study which was conducted in several countries that demonstrated that Women of reproductive age frequently experience anemia, which is a problem that depends on a number of factors and obviously changes through time and geographic location [19, 20]. Additionally, this regional difference in anemia may be linked to variations in the prevalence and distribution of infectious diseases, which frequently affect developing nations.

This study also, demonstrated that 24.6% of women had anemia. This finding was nearly similar with the study which was conducted in Ethiopia (24.2%) [21] and Serbian (27.7%) [22]. The finding of this study was lower than the study which was conducted in south eastern Nigeria(40.4%) [23], in Nepal (41%) [24], in Sudan (35.6%) [25], in east Africa(34.85%) [26], in Lao PDR (39.2%) [27] and in Pakistan(61.3% ) [28]. In contrast to this, the findings of this study higher than the study which was conducted in Rwanda(19.2%) [29], rural areas of Tabas (13.8%) [30], in south west Ethiopia (16.1%) [2] and in southwest china(18.9%) [31]. This variation could be because of the difference in socioeconomic, educational status and dietary habit [32]. In addition to this, we speculate that these variations might be exist due to variations in study area, study period, study population, sample size, geographic differences in prevalence of infectious disease and malnutrition, deep rooted cultural belief and taboo related to feeding habit. In addition to this, Our findings demonstrate that anemia among women of reproductive age is a moderate public health problem in Nigeria because anemia prevalence between the range of 20% and 40% considered as a moderate public health problem [3].

The multivariable logistic regression analysis also revealed that age groups, educational status, sex of household head, wealth index and frequency of listening radio were significantly associated with anemia among reproductive age women. These findings are also in agreement with a multi-nation study conducted in low and middle income countries (LMICs) that found that factors such as a woman’s wealth status, level of education, and behavior were significant predictors of anemia in reproductive age women [33].

According to this study’s findings, Women aged 30–34 years had a lower likelihood of being anemic compared with women aged between 15 years and 19 years. This finding was supported by the study which was conducted in in Nepal [34], and in Ethiopia [35]. This may be because women under the age of 19 are more likely to become pregnant unintentionally and experience pregnancy-related complications like severe preeclampsia, eclampsia, postpartum hemorrhage, poor fetal growth, and fetal distress, which raises the risk of anemia in this age group [36].

This study revealed that women of reproductive age who were attending in higher education had a lower likelihood of having anemia than those who were not attending education. This finding was supported by the study which was conducted in Ethiopia [37], in Nigeria [38], and Timor-Leste [39]. The first possibility is that women of reproductive age who were attending higher education were more knowledgeable about receiving antenatal care, the importance of iron-folate supplementation, the importance of dietary protein, intake of green leafy vegetables for the prevention of anemia [40], The second possibility were, when education levels rise, wealth and prestige tend to rise as well, and the desire to limit family size by utilizing modern contraceptives would increase lower the risk of anemia [41]. This is because women who use modern contraceptive methods avoid unintended pregnancy and childbirth-related complications, which may gradually lower the prevalence of anemia brought on by frequent blood loss. Another reasonable argument is that utilizing hormonal contraceptives, in particular, could lessen menstrual bleeding and decrease their vulnerability to anemia [42, 43]. The third possibility is that women who have higher levels of education tend to practice better nutrition and hygiene, which lowers their risk of anemia [44].

According to this analysis, households headed by men were less likely than those headed by women to have anemia. This finding was concurrent with the study which was conducted in Ethiopia [45], and in Nepal [46]. This may be the situation, when a woman takes over all of the household’s duties, both inside and outside the home, in the absence of the spouse. In this condition, these women tend to concentrate more on job outside the home and frequently allot less time to prepare and consume a range of foods in the home. This was in consistent with the study’s finding, which show that households headed by women have lower dietary quality than those headed by men [47]. In addition to this, anemia awareness and treatment seeking behavior was markedly lower in female-headed households than male-headed households [48]. In addition to this modern contraceptive utilization among male headed household were higher than female headed households that reduce the risk of anemia.

This study revealed that anemia was more prevalent in poorer women than in richest women. This finding was supported by the study which was conducted in Rwanda [49], in Myanmar [50], in Ghana [51], in Benin [52] and in Ethiopia [53]. This might be because mothers from rich households had a great opportunity to have a balanced diet in terms of meal frequency and variety of food [54], In addition to this the level of household wealth has a significant impact on access to education, basic healthcare services, health information [55] and better sanitation [48]. According to this study, anemia was more prevalent among women who listened to the radio less frequently than once a week than among those who did so at least once a week. This finding supported by the study which was conducted in Lao PDR [27] and Nigeria [38]. This might be that access to media is associated with awareness of the value and availability of health services, information on diets and nutrition, and information about contraceptive services. All are contributing factors in prevention and reduction of anemia.

## Conclusion

In this study Individual level factors were associated with anemia and also there were spatial variations in anemia across the region among reproductive-age women. Empowering women to have better educational status, improving the wealth index, and promoting education about prevention and control strategies of anemia in developing regions were the key factors to reduce anemia among reproductive age women in Nigeria.

### Strengths and limitations of this study

- The MIS has a similar design, with identical variables in a different environment; the result may, therefore, be applicable to other similar locations.
- The study used a sufficiently large sample size at the national level to ensure its representativeness.
- Recall bias is one of the potential drawbacks, especially for retrospective data based on past experiences.
- The magnitude of the bias is often unknown and correcting for the bias is difficult.
- Since, this study was cross sectional study, it doesn’t showed temporal relationships between independent and dependent variable.

## Data Availability

The data were obtained from Nigeria malaria indicators survey 2022 that was found at DHS portal of (https://dhsprogram.com/data/dataset_admin/index.cfm).
